# Visceral metastases, platelet dynamics, and PSA decline: from biomarkers to better outcomes in [^177^Lu]Lu‑PSMA‑617 therapy in metastatic castration-resistant prostate cancer

**DOI:** 10.7150/thno.109860

**Published:** 2025-03-03

**Authors:** Nathan Poterszman, Cécile Hammer-Lefevre, Clémence Porot, Julien Salvadori, Philippe Barthélémy, François Somme

**Affiliations:** 1 Nuclear Medicine and Molecular Imaging, Institut de Cancérologie Strasbourg Europe, Strasbourg, France.; 2 Radiopharmacy, Institut de Cancérologie Strasbourg Europe, Strasbourg, France.; 3 Radiophysics, Institut de Cancérologie Strasbourg Europe, Strasbourg, France.; 4 Oncology, Institut de Cancérologie Strasbourg Europe, Strasbourg, France.

**Keywords:** radioligand therapy, [^177^Lu]Lu-PSMA-617, metastatic castration-resistant prostate cancer (mCRPC), predictive factors, personalized oncology

## Abstract

Radioligand therapy with [^177^Lu]Lu-PSMA-617 constitutes a significant breakthrough in the management of metastatic castration-resistant prostate cancer (mCRPC). However, clinical outcomes remain variable due to the heterogeneity of patient profiles and limitations in current selection criteria. This study aims to identify simple and reproducible predictive factors to optimize therapy and improve patient stratification.

**Methods:** A retrospective analysis was conducted on 109 mCRPC patients treated with [^177^Lu]Lu-PSMA-617 at the Institut de Cancérologie Strasbourg Europe. Clinical, biological, and imaging parameters were collected, including early biological changes after the first cycle. Survival analyses and regression models were employed to identify prognostic and predictive factors.

**Results:** Visceral metastases (p < 0.001) and a decrease in platelet count above 25% (p = 0.004) between the first and second cycles were significantly associated with reduced overall survival. Furthermore, a decline in PSA levels above 30% following two cycles emerged as a robust predictor of treatment response, markedly affecting both biochemical progression-free survival (p < 0.001) and radiological progression-free survival (p = 0.002).

**Conclusion:** By combining three factors—visceral metastases, platelet count variation, and PSA reduction—patients could be stratified into distinct prognostic groups as early as after the first cycle. These findings pave the way for personalized management and enhanced clinical outcomes for mCRPC patients receiving [^177^Lu]Lu-PSMA-617.

## Introduction

Following the promising results of the VISION trial 1, radioligand therapy with [^177^Lu]Lu-PSMA-617 has been rapidly integrated into clinical practice, representing a significant advancement in the management of metastatic castration-resistant prostate cancer (mCRPC). This therapeutic approach offers a novel option for a disease characterized by high morbidity and limited treatment alternatives in advanced stages. However, despite its widespread adoption, several challenges persist, particularly regarding patient selection and post-treatment monitoring 2,3.

The sole mandatory eligibility criterion for this therapy is the presence of metastatic lesions with radiotracer uptake on PSMA PET/CT exceeding liver uptake, with the intensity of uptake showing strong predictive value 4. While this broad inclusion criterion ensures access to a large patient population, it may also contribute to early treatment failure in some cases, highlighting the need for a more refined patient stratification process. In real-world clinical settings, the heterogeneity in pathological presentations and prior treatment histories further complicates the identification of patients likely to derive meaningful and sustained benefits from this innovative therapy.

This study aims to identify simple and reproducible predictive and prognostic factors to optimize the use of [^177^Lu]Lu-PSMA-617 therapy. By focusing on a real-world cohort of mCRPC patients, it seeks to provide practical tools for clinicians to better select patients and tailor therapeutic strategies based on specific clinical, biological, and radiological characteristics. Particular attention is paid to parameters that significantly influence overall survival, biochemical progression-free survival, and radiological progression-free survival, with the goal of improving personalized patient management in this complex disease setting.

## Methods

### Patient population

We conducted a retrospective analysis of all patients harboring a mCRPC and treated with [^177^Lu]Lu-PSMA-617 at our institution Institut de Cancérologie Strasbourg Europe (ICANS). All patients had previously received at least one line of androgen receptor pathway inhibitor (ARPI) and one line of taxane-based chemotherapy, in accordance with the VISION trial and the French early access program. Patients with PSMA-positive lesions (with a majority of lesions harboring an uptake above liver level) on a pre-therapeutic [^68^Ga]Ga-PSMA-11 PET/CT were treated. Various clinical, biological, and radiological parameters were collected. These included the ISUP grade, the date of prostate cancer diagnosis, and all prior lines of systemic therapies administered in the mCRPC setting. The interval between the initial prostate cancer diagnosis and the first administration of [^177^Lu]Lu-PSMA-617 was calculated.

We did not perform mismatch exclusion using FDG PET/CT. This approach was applied only to a single patient with neurofibromatosis and known lymph node involvement, to differentiate between lesions arising from prostate cancer and those associated with neurofibromatosis. This strategy is based on three key arguments. First, in the VISION trial that validated [^177^Lu]Lu-PSMA-617, [18F]FDG PET/CT was not part of the inclusion criteria. Second, we aimed to streamline the patient workflow and avoid delays caused by scheduling an additional examination. Lastly, Seifert et al. published a study demonstrating that FDG/PSMA mismatch findings undetectable by PSMA PET alone occurred in only 3% of cases in a retrospective analysis of 89 VISION-like patients 5.

The institutional review board (Commission d'Orientation Recherche et Enseignement of the Institut de Cancérologie de Strasbourg) has approved the study (study number 2025_03). Informed consent was obtained from all participants. The need for ethics approval and consent to participate was waived. All research was performed in accordance with relevant guidelines and in accordance with the Declaration of Helsinki.

### Treatment protocol

Patients were treated up to 6 cycles of 7.4 GBq of [^177^Lu]Lu-PSMA-617 every 6 weeks according to VISION trial protocol. Treatment was disrupted in case of death, clinical, biological or radiological progression. For each cycle, data were gathered on *performance status*, weight, hemoglobin levels, platelet counts, alkaline phosphatase, albumin, renal clearance, PSA levels, and the administered activity of [^177^Lu]Lu-PSMA-617. Changes in hemoglobin levels, platelet counts, and PSA values between the first and second cycles were calculated. Additionally, for patients who received four or more cycles, the change in PSA levels between the first and fourth cycles was also determined. Bone scan and computed tomography were performed every two cycles.

### Image analysis

Patients were classified as M1a, M1b, or M1c based on pre-treatment staging using [^68^Ga]Ga-PSMA-11 PET/CT, in accordance with the PROMISE version 2 criteria. The maximum and peak standardized uptake values (SUVmax and SUVpeak) of the most intense radiotracer uptake in lymph node, bone, and visceral metastases were recorded for each patient.

### Outcomes

Patients were defined as responders if they met one of the following criteria: receiving at least five cycles of treatment or achieving a PSA reduction greater than 50% by the fourth cycle (consistent with the PCWG3 criteria for biochemical response). Biochemical progression-free survival (bPFS) was defined as the time from treatment initiation to a PSA increase exceeding 25%, as per PCWG3 criteria. Radiological progression-free survival (rPFS) was defined as the time from treatment initiation to disease progression observed on CT imaging (RECIST 1.1) or bone scintigraphy (PCWG3). Overall survival (OS) was defined as the time from treatment initiation to patient death from all cause.

### Statistical analysis

Demographic and clinical characteristics, laboratory values, and posttreatment outcomes were reviewed and compared. RECIST 1.1, PCWG3 and the Common Terminology Criteria for Adverse Events, version 5.0, were used to evaluate radiographic response and to grade treatment toxicities, respectively. Univariate and multivariate cox regressions were made to evaluate predictive and prognostic factors. The Kaplan-Meier method was used to estimate event time distributions, and log-rank tests were used for group comparisons. Hazard ratios were calculated with a 95% confidence interval. A p value of less than 0.05 was considered statistically significant. Statistical analyses were done using MedCalc version 23.0.8 and GraphPad Prism version 9.0.0.

## Results

### Patient population

Between January 1, 2022, and December 31, 2024, 163 patients initiated treatment with [^177^Lu]Lu-PSMA, for a total of 626 cycles. Thirty (18.4%) patients were enrolled in clinical research trials and 24 patients were actively undergoing treatment at the time of analysis. Therefore, one hundred nine patients constituted the analyzed cohort.

Of the 109 patients analyzed, 86 (78.9%) had received at least four prior lines of systemic therapies in the metastatic setting (range 3-12). Patients received a median of four cycles of [^177^Lu]Lu-PSMA-617, with a mean injected activity of 6.96 GBq per cycle.

On the pre-treatment [^68^Ga]Ga-PSMA-11 PET/CT imaging, 9 patients (8.3%) presented with lymph node-only involvement, 65 (59.6%) had bone metastases with or without lymph node metastases, and 35 (32.1%) exhibited visceral metastases, all of whom also had bone or lymph node involvement. The others clinical, biological, and radiological parameters are summarized in Table 1.

Of the 109 patients, 43 completed six cycles of therapy. The reasons for treatment discontinuation in the remaining 66 patients are detailed in Table 2. At the time of analysis, 70 (64.2%) patients had died, while 39 (35.8%) were still alive. The median overall survival of our cohort was 10.7 months with a median follow-up of 8.7 months. Regarding subsequent treatment, 26 patients underwent rechallenge with an androgen receptor pathway inhibitor, 21 received chemotherapy, and 6 underwent rechallenge with [177Lu]Lu-PSMA-617 (following a variable period without disease progression). Additionally, 1 patient was treated with radium-223. Fifty-five patients did not receive further treatment. Among these 55 patients, 39 experienced rapid disease progression leading to death shortly after the last infusion or had hematotoxicity precluding further treatment, whereas 16 remained under surveillance with stable disease. These data are summarized in Table 3.

### Prognostic factors

The first part of our analysis focused on identifying prognostic factors by evaluating parameters correlated with overall survival. In the univariate analysis, the following parameters were significantly associated with survival: the SUVmax of lymph node involvement (p = 0.039), the presence of visceral metastases (p < 0.001), hemoglobin levels at cycle 1 (C1) (p = 0.003), alkaline phosphatase levels at C1 (p < 0.001), the variation in platelet count between C1 and cycle 2 (C2) (p = 0.037), the variation in PSA levels between C1 and C2 (p = 0.02), and the time from initial diagnosis to the introduction of [^177^Lu]Lu-PSMA-617 (p = 0.024).

In the multivariate analysis, only the presence of visceral metastases (p < 0.001) and the variation in platelet count between C1 and C2 (p = 0.007) remained significantly associated with a shorter overall survival (C-index 0.82; 95% CI, 0.758-0.881).

**Figure 1:**
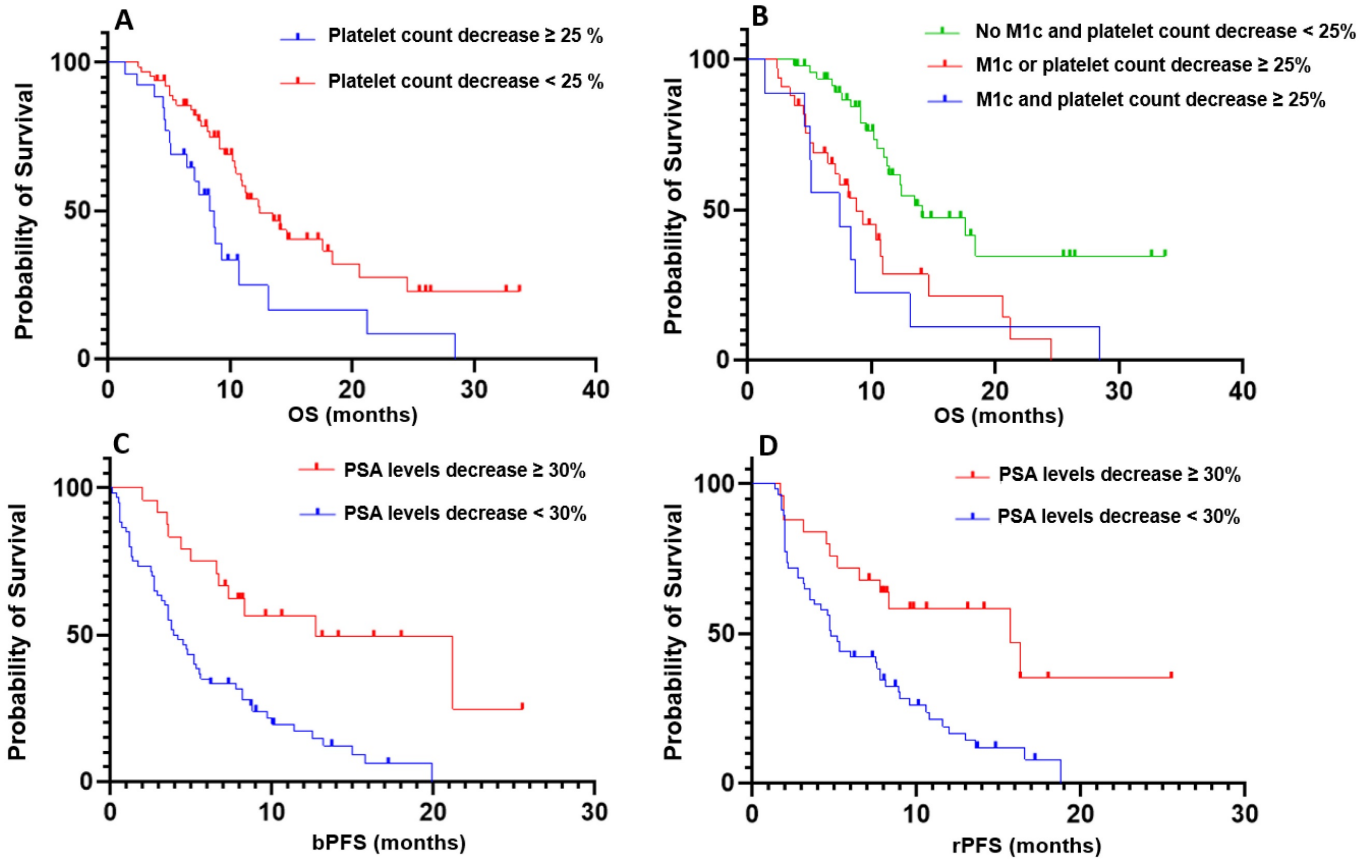
Kaplan-Meier curves illustrating prognostic and predictive parameters. **A**: Prognostic significance of platelet count variation with a threshold of 25%. **B**: Prognostic significance of the composite criterion combining the presence of visceral metastases and platelet count variation. **C:** Predictive significance of PSA level variation with a threshold of 30% for biochemical progression-free survival (bPFS). **D**: Predictive significance of PSA level variation with a threshold of 30% for radiological progression-free survival (rPFS).

Subsequently, we further explored the variation in platelet count between C1 and C2 to stratify patients into two distinct populations. A decreased platelet count above 25% differentiated the groups, revealing a significant difference in overall survival (p = 0.004; HR = 2.218; 95% CI, 1.133-4.341; Figure 1). Notably, only 16 patients developed grade 1 thrombocytopenia, and 1 patient experienced grade 3 thrombocytopenia at the second cycle of treatment.

Based on these findings, we combined the two criteria—presence of visceral metastases and a decrease in platelet count exceeding 25%—to further stratify patients according to overall survival. Patients without visceral metastases and without a platelet count decrease greater than 25% demonstrated significantly longer OS compared to patients with both factors (p < 0.001; HR = 3.51; 95% CI, 1.112-11.07; Figure 1), as well as compared to patients meeting only one of the two criteria (p < 0.001; HR = 2.673; 95% CI, 1.394-5.214; Figure 1). However, no significant difference in OS was observed between patients with both criteria and those meeting only one of the two criteria (p = 0.69).

### Predictive factors

The same methodology was applied to evaluate these parameters in relation to treatment response, assessed through both biochemical progression-free survival and radiological progression-free survival.

In the multivariate analysis, only the variation in PSA levels between C1 and C2 remained significantly associated with both biological progression-free survival (p < 0.001; C-index 0.748; 95% CI, 0.721-0.848) and rPFS (p = 0.002; C-index 0.751; 95% CI, 0.676-0.827). A decrease value above 30% effectively stratified patients into two distinct populations, demonstrating a significant difference in terms of bPFS (p < 0.001; HR = 3.01; 95% CI, 1.835-4.939; Figure 1) and radiological progression-free survival (p = 0.002; HR = 2.605; 95% CI, 1.557-4.359; Figure 1).

To assess the potential presence of a flare-up effect, we analyzed the correlation between the variation in PSA levels between C1 and C2 and the classification of patients as responders or non-responders. In detail, 46 patients received five or more cycles of treatment. Three patients completed only four cycles but achieved a PSA reduction greater than 50% at the fourth cycle. Among these, two patients demonstrated a complete response on [^68^Ga]Ga-PSMA-11 PET/CT with PSA reductions of 100% and 99.9%, respectively, and were appropriately classified as responders. However, the third patient, with a PSA reduction of 92.8%, was misclassified as a responder and later exhibited progression with evidence of a neuroendocrine component. Consequently, 60 patients were classified as non-responders and 49 as responders. The variation in PSA levels between C1 and C2 was significantly associated with the classification of patients as responders or non‑responders (p = 0.002).

### Comprehensive predictive model

**Figure 2:**
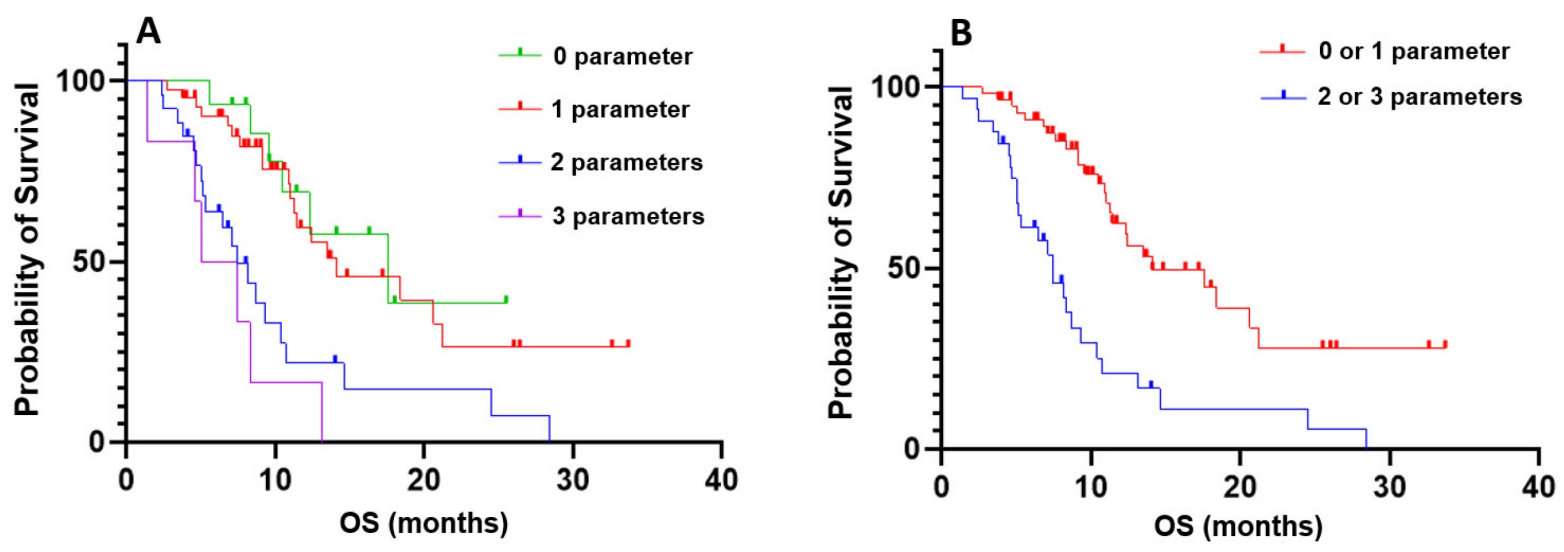
Kaplan-Meier curves of combined prognostic and predictive criteria. **A**: Stratification based on the combination of three criteria: absence of visceral metastases, a decrease in platelet count between the first and second cycles of more than 25%, and a decrease in PSA levels between the first and second cycles of less than 30%. Groups were defined as follows: 0 parameters (none of the criteria), 1 parameter (one of the criteria met), 2 parameters (two of the criteria met), and 3 parameters (all criteria met); **B**: Comparison of overall survival between patients with 0 or 1 parameter versus those with 2 or 3 parameters.

Finally, we combined the three parameters—the presence of visceral metastases, the variation in platelet count between C1 and C2 with a cutoff value of 25%, and the variation in PSA levels between C1 and C2 with a cutoff value of 30%—to further stratify patients by overall survival (OS). No significant difference in OS was observed between patients with none of the criteria and those with one criterion, nor between patients with two criteria and those with three criteria. However, patients with none or one criterion had significantly longer OS compared to those with two or three criteria (p < 0.001; HR = 3.216; 95% IC, 1.698 - 6.092; Figure 2).

## Discussion

We aimed to identify simple and reproducible clinical, biological, and radiological parameters to facilitate the early identification of patients likely to benefit from [^177^Lu]Lu-PSMA-617 therapy. Our findings demonstrated that the presence of visceral metastases and the variation in platelet count between the first and second treatment cycles are two strong prognostic factors for patients undergoing radioligand therapy. Additionally, the variation in PSA levels between C1 and C2 emerged as a robust predictive factor of treatment response, influencing both biochemical progression-free survival and radiological progression-free survival.

By combining these three parameters—the presence of visceral metastases, the variation in platelet count between C1 and C2, and the variation in PSA levels between C1 and C2—we successfully stratified patients into two distinct prognostic groups. Patients with none or only one unfavorable parameter had significantly longer overall survival compared to those with two or three unfavorable parameters, the latter reflecting an increased likelihood of early treatment failure. Importantly, these parameters are readily available by the second treatment cycle, enabling clinicians to closely monitor and adjust the management of those patients.

When managing patients who demonstrate a suboptimal response after the first cycle of [^177^Lu]Lu-PSMA-617 therapy, a structured, evidence-based approach is essential. Our findings highlight three key prognostic factors which are strongly associated with treatment outcomes. Given the potential limitations of PSA kinetics 6 and potential late responders 7, therapeutic modifications should not be based solely on early PSA changes. Instead, response evaluation should integrate clinical, biological, and imaging parameters. If at least two of the three unfavorable criteria are met after the first cycle, reassessment of the therapeutic strategy should be considered. This may include closer monitoring, additional imaging to exclude disease progression, or even a switch to an alternative therapy.

The presence of visceral metastases was already confirmed as a poor prognostic factor for patients with metastatic castration-resistant prostate cancer treated with [^177^Lu]Lu-PSMA-617. In a meta-analysis of 12 articles including 1504 patients, the presence of visceral metastases was associated with poor response and survival outcomes in patients of mCRPC treated with Lu-PSMA radioligand therapy (pooled multivariate HR, 2.22; 95% CI, 1.82-2.70) 8. This was especially true for patients with liver metastasis. Muniz *et al.* analyzed 273 mCRPC patients, with 43 of them harboring liver metastasis treated with [^177^Lu]Lu-PSMA-617. They reported that on multivariate analysis, the presence of liver metastasis was independently associated with shorter survival (HR, 4.06; p < 0.001) 9. Our results reveal that the presence of only one of the three prognostic factors is not sufficient to significantly worsen patient outcomes. This suggests that patients with visceral metastases detected on the baseline PSMA PET/CT should still be considered for [^177^Lu]Lu-PSMA-617 as a viable therapeutic option. However, close monitoring of these patients is crucial, particularly in assessing the early evolution of biological markers such as platelet counts and PSA levels after the second treatment cycle. This approach is essential to identify those with an unfavorable prognosis and, if necessary, facilitate an early switch to an alternative therapeutic strategy.

A decrease in platelet count between the first and second cycles of therapy was significantly associated with shorter OS in our cohort. Among 27 patients with an early platelet decline above 25%, only one patient developed grade 3 thrombocytopenia, while 16 had grade 1, and 10 still had normal platelet counts. Thus, the trend of platelet decline between cycles appears more critical than the absolute count, potentially reflecting early bone marrow toxicity that could predispose to subsequent treatment failure. Notably, cabazitaxel, the typical therapeutic alternative following treatment with [^177^Lu]Lu-PSMA-617, is unlikely to be administered if platelet counts do not exceed 100 G/L. This bone marrow sensitivity is likely multifactorial, influenced by both prior treatments and radioligand therapy induced bone marrow irradiation particularly in patients with extensive bone metastases. In a multicenter study in patients with a diffuse bone marrow involvment, Gafita *et al.* reported higher rates of grade 3 adverse events that in VISION trial, especially for anemia and thrombocytopenia 10. In their real-world cohort, Tuchayi *et al.* reported an average decrease in platelet count of 42%, with 21 patients experiencing grade 3 or 4 thrombocytopenia 11. Similarly, in the large prospective REALITY registry, the authors observed grade 3/4 thrombocytopenia in 4.3% of patients and grade 1/2 in 18.1% 12. From an oncologic perspective, we contend that grade 1 and 2 thrombocytopenia may be nearly as detrimental as higher grades. We did not observe a systematic switch to another treatment due to thrombocytopenia. However, further studies are needed to determine whether this early platelet decrease is specifically related to radioligand therapy toxicity or represents a more general marker of impaired bone marrow reserve affecting overall treatment sequencing.

The observation that the combination of visceral metastases and a significant platelet decline (>25%) between the first and second treatment cycles does not further worsen prognosis compared to either factor alone could be explained by several underlying mechanisms. First, patients with visceral metastases often exhibit an aggressive disease phenotype 13, which is associated with systemic deterioration, including potential bone marrow impairment. A concurrent decline in platelet count may reflect an advanced disease state rather than exerting an independent additional impact on prognosis. Moreover, the presence of visceral metastases is already a strong predictor of poor overall survival 14, suggesting that further worsening of a specific hematological parameter may not significantly alter an already limited life expectancy. This phenomenon is commonly observed in oncology, where multiple adverse factors may not always have strictly additive effects but rather indicate a critical disease burden beyond which additional prognostic factors become less relevant. Lastly, the baseline bone marrow involvement was not mesured and could contribute to this finding. Rather than representing a single converging mechanism, we believe that visceral metastases and early platelet decline are two distinct prognostic factors reflecting different aspects of disease progression. Visceral metastases primarily indicate aggressive tumor biology and metastatic spread, whereas platelet decline likely reflects treatment-related bone marrow vulnerability and preexisting hematopoietic fragility. While both factors independently correlate with worse survival, their effects do not appear to be strictly additive, reinforcing the need for a multidimensional approach when assessing early treatment prognosis in patients receiving [^177^Lu]Lu-PSMA-617.

Lastly, the variation in PSA levels between C1 and C2 was strongly predictive of response to radioligand therapy. The optimal follow-up management for patients undergoing [^177^Lu]Lu-PSMA-617 therapy remains a subject of ongoing debate. In our cohort, this parameter was significantly correlated with both biochemical progression-free survival (defined as a PSA increase of more than 25% from the nadir) and radiological progression-free survival, emphasizing its utility in patient monitoring. The PCWG3 criteria recommend using a PSA reduction of over 50% to define a PSA response 15. However, a reduction of over 30% is also frequently employed to assess an early response and evaluate partial treatment efficacy. This threshold has been shown to correlate with survival outcomes 16. In a large retrospective observational study including 233 patients with mCRPC treated with [^177^Lu]Lu-PSMA, Kafka *et al.* presented similar results to ours 17. They reported that a PSA decrease ≥ 30% after the first two cycles was significantly correlated to treatment reponse. The primary limitation of relying on an early PSA reduction is the potential for a flare-up effect. This was limited by the persistent correlation between the variation of the PSA and the classification of patients as responders or non‑responders as mentioned above. Finally, PSA alone may have limitations in monitoring mCRPC, particularly due to its potential discordance with interim PSMA PET/CT findings. Prasad *et al.* analyzed 38 mCRPC patients treated with at least two cycles of radioligand therapy and found that PSA had only a fair level of agreement with PSMA PET/CT, indicating limited predictive value 18. Similar findings have been reported in patients receiving androgen receptor pathway inhibitors or chemotherapy 19. Kleiburg *et al.* further demonstrated that among patients with a PSA decline >50%, 31% exhibited progressive disease on PSMA PET/CT, which was associated with an increased risk of mortality. Thus, as emphasized in our article, integrating the three key parameters reported is essential for optimal patient management.

Several nomograms have been developed to evaluate models predicting outcomes in patients with mCRPC or those treated with [^177^Lu]Lu-PSMA-617. The first nomogram was developed by Gafita *et al.*, incorporating predictors such as time since the initial diagnosis of prostate cancer, chemotherapy status, baseline hemoglobin concentration, and [^68^Ga]Ga-PSMA-11 PET/CT parameters (molecular imaging TNM classification and tumor burden). The C‑indices for the OS and rPFS models were 0.71 (95% CI, 0.69-0.73) and 0.70 (95% CI, 0.68-0.72), respectively. This was based on a cohort of 270 patients, divided into a development set (n = 196) and a validation set (n = 74). Low-risk patients demonstrated significantly longer OS (24.9 months [95% CI, 16.8-27.3] vs. 7.4 months [4.0-10.8]; p < 0.0001) and PSA progression-free survival (6.6 months [6.0-7.1] vs. 2.5 months [1.2-3.8]; p = 0.022) compared to high-risk patients 20. Karpinski *et al.* integrated PSMA PET/CT imaging with the PROMISE criteria to classify patients into low- and high-risk groups based on survival outcomes 21. Their study included 2 414 patients across various stages of the disease. Key predictors of survival identified were locoregional lymph node metastases, distant metastases, tumor volume, tumor mean standardized uptake value, and total tumor lesion count. Specifically, in the mCRPC cohort, the authors analyzed 270 patients and demonstrated that their nomograms effectively stratified patients into two survival groups. No significant differences were observed when comparing their model to the GAFITA nomogram at this stage (area under the curve: 0.71 vs. 0.75; p = 0.23). More recently, Herrmann *et al.* conducted a post hoc analysis of the VISION trial to construct multivariable models for patient outcomes 22. Using data from 551 patients in the [^177^Lu]Lu-PSMA-617 treatment arm, they identified multiple parameters associated with OS and rPFS. These parameters included whole-body maximum standardized uptake value, time since diagnosis, opioid analgesic use, aspartate aminotransferase levels, hemoglobin, lymphocyte count, the presence of PSMA-positive lesions in lymph nodes, liver metastases detected on computed tomography, lactate dehydrogenase, alkaline phosphatase, and neutrophil count. Their nomograms were strongly correlated with OS (C‑index: 0.73; 95% CI: 0.70-0.76) and rPFS (C-index: 0.68; 95% CI: 0.65-0.72). Nevertheless, these tools are rarely implemented in routine clinical practice due to the extensive number of variables required for their application.

The main limitation of this study lies in its retrospective design, which may introduce selection bias. Indeed, data were collected retrospectively from existing medical records, which does not allow for the control of certain potential factors that may have influenced treatment outcomes. Although all patients received at least one line of androgen receptor pathway inhibitor and one line of taxane-based chemotherapy, there was significant heterogeneity in the total number of prior treatment lines received (ranging from 3 to 12 lines). This could introduce variability in the response to [^177^Lu]Lu-PSMA-617 treatment, making it more difficult to directly compare outcomes between patients with very different treatment histories. This study evaluated short-term prognostic factors based on overall survival and disease progression, data on the long-term effects of radioligand therapy, such as late side effects or cumulative toxicity, are limited. Longer-term follow-up would be necessary to assess the long-term impacts of this treatment and to determine whether there are late-onset adverse effects that were not captured in this study, by analogy with the estimated long-term toxicity of [^177^Lu]Lu-DOTATATE 23.

Lastly, the decision to use CT and bone scans instead of PSMA-PET/CT for interim staging in our study was primarily influenced by practical considerations rather than a dismissal of the superior sensitivity of PSMA-PET/CT, as demonstrated with RECIP criteria 24. First, in ongoing trials investigating [^177^Lu]Lu-PSMA therapy—such as PSMAddition (NCT04720157), ECLIPSE (NCT05204927), and PSMAfore (NCT04689828)—CT and bone scans remain the imaging modalities of choice, as was the case in the VISION trial. Secondly, their use facilitates comparability with historical datasets and real-world clinical decision-making frameworks, which have predominantly relied on these methods. The incorporation of interim PSMA PET/CT could enhance response assessment by detecting early molecular changes before morphological progression becomes apparent on conventional imaging. This could enable more refined stratification of responders and non-responders, potentially leading to earlier therapy modifications or discontinuation in cases of clear progression. However, as our findings suggest, a combination of clinical, biological, and imaging parameters—including visceral metastases, platelet count variations, and PSA dynamics—already allows for early risk stratification. Future prospective studies evaluating the added value of interim PSMA-PET/CT in guiding treatment adaptations within this framework would be necessary to establish its routine use in this specific context.

## Conclusion

Our results demonstrate that patients can be stratified into two groups with distinct outcomes as early as after the first cycle of [^177^Lu]Lu-PSMA-617, with three simple clinical and biological factors. These findings pave the way for personalized management and could improve outcomes for patients undergoing [^177^Lu]Lu-PSMA-617 therapy.

## Figures and Tables

**Table 1:** Baseline clinical, biological, and radiological data of the analyzed patients were summarized. Abbreviations: RLT, radioligand therapy; C1, first cycle of [^177^Lu]Lu-PSMA-617; C2, second cycle of [^177^Lu]Lu-PSMA-617; SUV, standardized uptake value

	Parameter	Median	Range
**Clinical**	ISUP grade	4	1 — 5
	Previous line of systemic therapies	4	3 — 12
	Age (years)	72	50 — 88
	Performance status	1	0 — 3
	Interval between diagnosis and RLT (months)	96.1	12.5 — 343.3
	Number of RLT cycles	4	1 — 6
**Biological**	Hemoglobin levels (g/dl)	11.4	6.6 — 15.3
	Platelet counts (G/l)	246	58 — 846
	Phosphatase alkaline (ui/l)	120	39 — 1693
	PSA levels (ng/ml)	102.65	0.006 — 4015
	Changes in hemoglobin levels C1—C2 (%)	-1.5	-36.7 — 24.7
	Changes in platelet counts C1—C2 (%)	-11.5	-62.8 — 104.8
	Changes in PSA levels C1—C2 (%)	-8.9	-95.8 — 1817.4
**PSMA PET/CT**	Lymph node metastasis SUVmax	31.2	3.5 — 230.2
	Lymph node metastasis SUVpeak	17.3	1.4 — 172.2
	Bone metastasis SUVmax	41	5.7 — 233.1
	Bone metastasis SUVpeak	26.2	2.5 — 140
	Visceral metastasis SUVmax	16.4	1.6 — 99.3
	Visceral metastasis SUVpeak	11.6	1 — 58.8

**Table 2:** Reasons for treatment discontinuation in the 66 patients who did not complete six cycles of therapy

Reasons for treatment discontinuation	n — %
Clinical progression	18 — 27.3%
Biological progression	4 — 6.1%
Radiological progression	11 — 16.7%
Clinical and biological progression	9 — 13.6%
Clinical and radiological progression	4 — 6.1%
Biological and radiological progression	20 — 30.3%

**Table 3:** Description of subsequent treatment after [^177^Lu]Lu-PSMA-617 therapy. Abbreviation: ARPI, androgen receptor pathway inhibitor

Subsequent treatment	n — %
None	55 — 50.5%
Death or haematotoxicity	39 — 35.8%
Stable disease under follow-up	16 — 14.7%
ARPI	26 — 23.9%
Chemotherapy	21 — 19.3%
Rechallenge of [^177^Lu]Lu-PSMA-617	6 — 5.5%
Radium 223	1 — 0.9%

## Abbreviations

ARPI: androgen receptor pathway inhibitor
bPFS: biochemical progression-free survival
CI: confidence interval
CTCAE: common terminology criteria for adverse events
HR: hazard ratio
ICANS: Institut de Cancérologie Strasbourg Europe
ISUP: international society of urological pathology
mCRPC: metastatic castration-resistant prostate cancer
OS: overall survival
PCWG3: prostate cancer working group 3
PET/CT: positron emission tomography/computed tomography
PSMA: prostate-specific membrane antigen
RECIST 1.1: response evaluation criteria in solid tumors, version 1.1
RLT: radioligand therapy
rPFS: radiological progression-free survival
SUVmax: maximum standardized uptake value
SUVpeak: peak standardized uptake value
